# Exploration of Likert scale in terms of continuous variable with parametric statistical methods

**DOI:** 10.1186/s12874-025-02668-1

**Published:** 2025-09-29

**Authors:** Iksoo Huh, Jungsoo Gim

**Affiliations:** 1 College of Nursing, Seoul National University, Seoul, Republic of Korea; 2 The Research Institute of Nursing Science, Seoul National University, Seoul, Republic of Korea; 3 Department of Biomedical Science, Chosun University, Gwangju, Republic of Korea; 4 Institute of Well-Aging Medicare & CSU G-LAMP Project Group, Chosun University, Gwangju, Republic of Korea; 5 BK FOUR Department of Integrative Biological Sciences, Chosun University, Gwangju, Republic of Korea

**Keywords:** Likert scale, Continuous variable, Statistical power, Correlation, Factor analysis, Structural equation model

## Abstract

**Background:**

The Likert scale is an ordinal variable that measures the intensity of responses from research participants. It is widely used not only in social sciences, such as sociology and psychology, but also in survey-based research fields, such as nursing and public health. Among the approaches for handling the Likert-scale data, treating it as a continuous variable has been commonly used because it facilitates the application of parametric statistical methods and interpretation of results. In addition, from a perspective of statistical principle, this type of approach has been widely discussed and considered unproblematic. However, studies exploring the characteristics of the Likert scale in the approach with simulations are relatively rare. Thus, this study aimed to confirm the validity of the approach with simulation that compared the statistical characteristics of the Likert scale variable with those of variables from an assumed continuous latent distribution.

**Methods:**

In the Monte Carlo simulation study, the rectified normal distribution was specifically assumed for the continuous latent distribution. Then, type 1 error, statistical power, and correlation were compared across various situations involving both single and multiple variables.

**Results:**

From the simulation study, we found that a Likert scale with five or more points exhibited statistical characteristics comparable to those of the variable derived from the latent continuous distribution.

**Conclusions:**

Based on the results, we confirmed that the Likert scale can be used as a continuous variable in various parametric statistical methods. The corresponding suggested guidelines are expected to assist researchers in research design, data analysis, and results interpretation.

## Background

The Likert scale (LKS), an ordinal variable widely used in survey-based research, was originally developed by Rensis Likert in 1932 [1]. Responses to questions that measure subjective opinions in an ordinal scale—such as satisfaction with restaurant services, preference for policies, or levels of depressive feelings—are considered forms of LKS. Given the increasing diversity of research fields focused on human responses [2, 3], the importance of this scale also continues to grow. Specifically, LKS is extensively used not only in social sciences such as sociology and psychology—Rensis Likert’s areas of interest [4–6]—but also in other survey-based fields such as nursing and public health. For example, numerous instruments have been developed to measure various aspects of the nursing environments [7–9], and to assess the quality of life in public health [10–12].

To analyze the surveyed data measured using the LKS, several approaches have been suggested, depending on the perspectives with which researchers interpret the data. First, when the LKS is considered only to have ordinal characteristics and not to have equal intervals among the response levels [13], nonparametric statistical methods such as Kendal’s tau [14] and parametric statistical methods such as the proportional odds model [15] have been used. By contrast, when the LKS is considered to have equal intervals among the levels [13], parametric statistical methods, including the t-test, ANOVA, and linear regression [16] have been applied, similar to the analyses with continuous variables.

Although the LKS with equal interval is easy to apply and interpret, the statistical feasibility of treating this discrete variable with a few levels as a continuous variable has been questioned and debated in several reviews [13, 16–18]. In these reviews, they generally recognized the feasibility of this approach, and the most cited one among them [16] concluded that most concerns regarding the approach with parametric methods are not critical in terms of the asymptotic statistical principles, including the central limit theorem [19]. Another review complemented the conclusion with more detailed suggestions for real data analysis [17]. Although these suggestions were useful, they were not confirmed through simulation analysis. Therefore, readers may find it difficult to understand their appropriateness based on quantifiable criteria, such as type 1 error and statistical power, in various situations. Thus, in our study, we strengthened the conclusions using Monte Carlo simulations designed for several possible situations. Specifically, we verified the conclusion by comparing the statistical properties of the continuous latent variable assumed for the benchmark and its transformed LKS variables. For the continuous latent variable, we used a rectified normal (RTN) distribution [20, 21] that can incorporate floor and ceiling effects [22] as an example. In addition to investigating type 1 error and statistical power, we examined whether the correlation structure was preserved when multiple RTN variables were transformed into multiple LKS variables, considering situations concerning factor analysis and structural equation modeling [23] which are commonly used in nursing research. Based on the simulation analysis results, which systematically and precisely quantified the statistical characteristics of the LKS data, we expect researchers to gain clearer insights for research design and greater confidence in their results when treating the LKS variable as a continuous variable, especially when the continuous latent distribution follows the normal distribution or its derived ones.

## Methods

### Assumption of continuous latent distribution of the LKS variable

In this study, we assumed that the LKS variables were transformed from the RTN variables as an example. The RTN distribution originates from an ordinary normal distribution [24]; however, its unique characteristic is that its range has upper and/or lower limits with probability masses. Therefore, the RTN distribution is easily generated from the ordinary normal distribution by setting limits and assigning probabilities outside these limits. This may be confused with the truncated normal distribution [25], as both have bounded range. However, the key difference between the RTN distribution and truncated normal distribution is that the truncated normal distribution has no probability masses at the limits. Instead, its overall density is inflated by the reciprocal value of the probability within the limits.

**Fig. 1:**
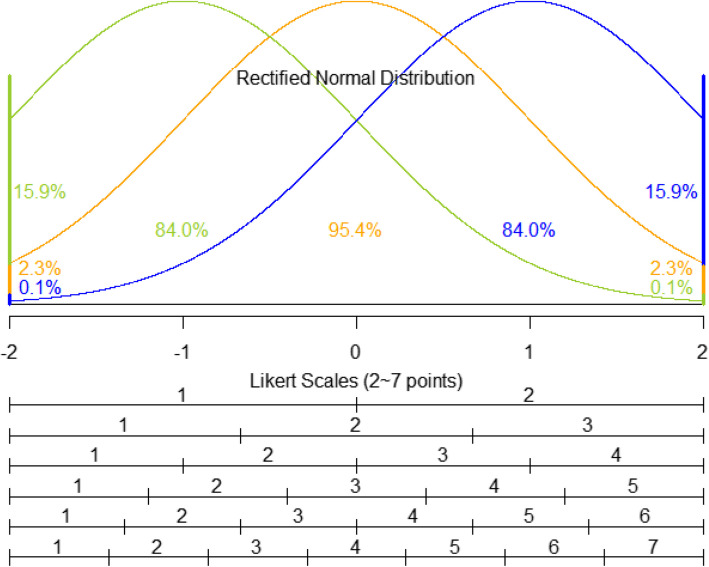
Visual description of the RTN distributions and their transformed LKS variables. The green, orange, and blue colored distributions have median values at −1, 0, and 1, respectively. Probabilities at the both limits and within the range are also provided. From a fixed RTN distribution, probability for each level of a certain type LKS variable is determined.

Specifically, we selected the RTN distribution as a continuous latent distribution because participants answering the LKS questions were required to select a level representing the intensity of their responses. In this context, the minimum and maximum levels of the LKS variable may cover a wider range of intensities than the other levels, which can be called floor and ceiling effects [22]. For example, when we test students in school examinations, the probabilities at the limits of the test score can vary according to the difficulty level of the test, and final grade may be determined based on the test score divided into intervals of the same length. Similarly, concerning the limit of the observable range, when surveying the restaurant preferences using a five-point LKS variable, some respondents may have extremely high preference, but they can select no higher than five, while others can choose no lower than one for the opposite reason. Therefore, we assumed the RTN distribution as the continuous latent distribution of response intensity. Then we generated the LKS variables by dividing the RTN distribution’s range into intervals of the same length and transforming these intervals into integers. For clarity, Fig. 1 shows the RTN distribution and its transformation into the LKS variables. In detail, we additionally assumed that the RTN distribution ranged between −2 and 2, covering 95.4% of the distribution when centered at z = 0. As the latent distribution shifts left or right, the probability masses at z = −2 or 2 change accordingly, and these shifts are ultimately reflected in the distribution of the LKS variables.

### Simulation settings in our study

In the previous section, we explained the characteristics of the RTN distribution and how we generated the LKS variables from it. For types of the LKS variables, we selected two to seven points, expecting that as the points increased, the statistical properties of the LKS variable would become more similar to those of the RTN variable. For statistical analysis, we first focused on the t-test to compare means between the two groups. For effect size in the t-test, we used the standardized mean difference $\frac{{\mu }_{1}-{\mu }_{2}}{\sqrt{({{n}_{2}\upsigma }_{1}^{2}+{n}_{1}{\upsigma }_{2}^{2})/({n}_{1}+{n}_{2})}}$, where ${\mu }_{i}$, ${n}_{i}$, and ${\upsigma }_{i}^{2}$ are mean, sample size, and variance of i^th^ group, respectively. This effect size considered heteroscedasticity [26] and is an extended version of Hedge’s *d* [27]. When the effect size was zero, it was for assessing type 1 error, while nonzero effect sizes were for statistical power. Moreover, in the specific comparison, we assumed that variable in each group was not only from a single variable itself, but also from a merged variable using multiple correlated variables, considering situations that many surveys contain multiple LKS-type questions, some of which are conceptually similar or were developed to measure a certain concept [7, 8], and the similar questions have often been merged into a new variable to obtain the total score. For this purpose, multiple RTN variables were generated using several pairwise Pearson correlations (= 0, 0.25, 0.5, 0.75, and 1). Subsequently, we converted the RTN variables into LKS variables and merged some numbers of them (= 2, 3, 5, and 10), and calculated statistical power for each combination of the two factors.

Second, for situations when multiple LKS variables can also be used in their original form in the factor analysis or structural equation model analysis [23], we evaluated preservation of the pairwise correlations during the variable type transformation with goodness of fit index (GFI) and standardized root mean squared residual (SRMR) [28] measures, because they can evaluate the similarity between the correlation matrices from the RTN and LKS variables without any additional model assumptions. Specifically, the GFI is given as 1- $\frac{\text{F}(\text{A},\text{B})}{\text{F}(\text{A},\text{I})}$, where $\text{F}\left(\text{A},\text{B}\right)$ is a difference measure between matrix A and B and I is the identity matrix, which evaluates the improved relative similarity between a reference and a target matrix compared to the similarity between the reference and the identity matrix. When the value is greater than 0.95, similarity is regarded as excellent. The SRMR is given as $\sqrt{2\times {\sum }_{i=1}^{p}{\sum }_{j=1}^{i}\frac{{({a}_{ij}-{b}_{ij})}^{2}}{p\times (p+1)}}$, where which ${a}_{ij}$ and ${b}_{ij}$ are the element of i^th^ row and j^th^ column of matrix A and B, respectively, and p is the number of variables. This measure evaluates the standardized root mean of squared differences between non-redundant elements of two correlation matrices, and a value under 0.05 is considered excellent.

Finally, we assessed the statistical power of linear regression according to various number of explanatory variables. For this purpose, we generated multiple RTN variables with pairwise correlation of 0.2 and selected one of them as a response variable and the others as explanatory variables. The number of explanatory variables was set to 1, 2, 3, 5, and 10. Subsequently, the RTN and its transformed LKS variables were assigned according to each type combination of explanatory and response variables. In this analysis, up to five points of the LKS variables were considered to reduce the number of combinations. Then, we conducted a linear regression and calculated the statistical power based on the *p*-value of the regression model.

Besides, for each setting of the Monte Carlo simulation, we performed 10,000 iterations, which could provide reasonably precise standard error for estimating proportions such as type 1 error and statistical power. In detail, the corresponding standard error is $\sqrt{p\times (1-p)/n}$ and it has the largest value of 0.5% at p = 0.5. With the standard error, 95% confidence interval of the proportions is derived as $\widehat{p}\pm 1.96\times \sqrt{\widehat{p}\times (1-\widehat{p})/n}$. We also provided required sample size in the LKS analysis results which can achieve same statistical power of the RTN variable. Based on these analysis results, we proposed statistical guidelines for the reliable use of the LKS variables.

## Results

Based on the schemes in the previous section, we conducted a simulation study using artificially generated datasets. First, we compared means between the two groups using the t-test. For effect size (ES), we set the values at 0, 0.2, and 0.5, where 0 indicates no effect for the estimation of type 1 error, while 0.2 and 0.5 represent small and moderate effects for the estimation of statistical power, respectively. For the sample sizes, we selected 30, 50, 100, 300, and 500. For each parameter setting, we generated 10,000 datasets and counted the number of significant results (*p* < 0.05) for type 1 errors and statistical power.

The detailed results are provided in Table 1. Considering 95% confidence interval, we found that most results for effect size of 0 captured the nominal significance level of 0.05, and no clear trend was observed based on the sample size or the LKS variable points. However, when the effect size was 0.2, statistical power was influenced by these two factors. Specifically, as the sample size increased, the statistical power also increased. Regarding the response type, the RTN variable exhibited the highest statistical power, while the two-point LKS variable had the lowest. Additionally, as the number of points in the LKS variable increased, its statistical power increased and approached that of the RTN variable. This suggests that the LKS variables lost the original information of the RTN variable during the transformation, but the amount of lost information decreased as the points of the LKS variable increased. Consequently, the statistical power of the LKS variable with five or more points can be considered similar to that of the RTN variable. For effect size of 0.5, the patterns observed with effect size of 0.2 became more evident. Between the two factors in the results from both nonzero effect size settings, sample size had a greater impact on statistical power than the type of variable. For example, when the sample size was 30 and the effect size was 0.2, the statistical power of the RTN variable was 0.1207 and that of the two-point LKS variable was 0.0933. However, when the sample size increased to 50, the statistical power of the two-point LKS variable increased to 0.1386. This compensatory effect was consistently observed across all non-zero effect size settings, indicating that although the RTN variable had the highest statistical power, increasing the sample size of the LKS variable might easily compensate for its loss.

**Table 1:** Power analysis of two sample t-test with the RTN variables and their transformed LKS variables. Proportions (%) of the significantly identified sets and their standard errors were provided.

Effect Size	Type of variable	Sample size per group				
		30	50	100	300	500
0	RTN	4.94 $\pm$ 0.22	5.00 $\pm$ 0.22	4.80 $\pm$ 0.21	5.01 $\pm$ 0.22	5.09 $\pm$ 0.22
	2-point LKS	4.86 $\pm$ 0.22	5.42 $\pm$ 0.23	5.46 $\pm$ 0.23	4.89 $\pm$ 0.22	5.43 $\pm$ 0.23
	3-point LKS	5.04 $\pm$ 0.22	4.95 $\pm$ 0.22	4.90 $\pm$ 0.22	5.00 $\pm$ 0.22	5.13 $\pm$ 0.22
	4-point LKS	4.90 $\pm$ 0.22	4.96 $\pm$ 0.22	4.58 $\pm$ 0.21	4.95 $\pm$ 0.22	5.16 $\pm$ 0.22
	5-point LKS	4.65 $\pm$ 0.21	5.30 $\pm$ 0.22	4.84 $\pm$ 0.21	5.09 $\pm$ 0.22	5.22 $\pm$ 0.22
	6-point LKS	4.91 $\pm$ 0.22	4.96 $\pm$ 0.22	4.89 $\pm$ 0.22	5.04 $\pm$ 0.22	4.92 $\pm$ 0.22
	7-point LKS	4.85 $\pm$ 0.21	4.75 $\pm$ 0.21	4.96 $\pm$ 0.22	5.16 $\pm$ 0.22	4.99 $\pm$ 0.22
0.2	RTN	12.07 $\pm$ 0.33	16.85 $\pm$ 0.37	29.19 $\pm$ 0.45	68.10 $\pm$ 0.47	87.39 $\pm$ 0.33
	2-point LKS	9.33 $\pm$ 0.29	13.86 $\pm$ 0.35	21.81 $\pm$ 0.41	50.12 $\pm$ 0.50	71.71 $\pm$ 0.45
	3-point LKS	10.75 $\pm$ 0.31	14.24 $\pm$ 0.35	24.58 $\pm$ 0.43	58.93 $\pm$ 0.49	80.29 $\pm$ 0.40
	4-point LKS	11.15 $\pm$ 0.31	15.91 $\pm$ 0.37	26.46 $\pm$ 0.44	62.57 $\pm$ 0.48	83.45 $\pm$ 0.37
	5-point LKS	11.64 $\pm$ 0.32	15.93 $\pm$ 0.37	27.33 $\pm$ 0.45	64.89 $\pm$ 0.48	85.19 $\pm$ 0.36
	6-point LKS	11.76 $\pm$ 0.32	16.12 $\pm$ 0.37	27.78 $\pm$ 0.45	65.28 $\pm$ 0.48	85.82 $\pm$ 0.35
	7-point LKS	11.74 $\pm$ 0.32	16.22 $\pm$ 0.37	28.10 $\pm$ 0.45	65.89 $\pm$ 0.47	85.96 $\pm$ 0.35
0.5	RTN	47.06 $\pm$ 0.50	69.41 $\pm$ 0.46	93.90 $\pm$ 0.24	99.99 $\pm$ 0.01	100.00 $\pm$ 0.00
	2-point LKS	32.31 $\pm$ 0.47	51.10 $\pm$ 0.50	80.02 $\pm$ 0.40	99.77 $\pm$ 0.05	100.00 $\pm$ 0.00
	3-point LKS	40.43 $\pm$ 0.49	60.57 $\pm$ 0.49	88.24 $\pm$ 0.32	99.97 $\pm$ 0.02	100.00 $\pm$ 0.00
	4-point LKS	42.20 $\pm$ 0.49	64.14 $\pm$ 0.48	90.83 $\pm$ 0.29	99.99 $\pm$ 0.01	100.00 $\pm$ 0.00
	5-point LKS	44.24 $\pm$ 0.50	66.38 $\pm$ 0.47	91.90 $\pm$ 0.27	99.99 $\pm$ 0.01	100.00 $\pm$ 0.00
	6-point LKS	44.88 $\pm$ 0.50	67.26 $\pm$ 0.47	92.54 $\pm$ 0.26	99.99 $\pm$ 0.01	100.00 $\pm$ 0.00
	7-point LKS	45.18 $\pm$ 0.50	67.99 $\pm$ 0.47	92.95 $\pm$ 0.26	99.99 $\pm$ 0.01	100.00 $\pm$ 0.00

Provided effect sizes were based on the RTN variables. Significant level was determined to be 0.05.

**Fig. 2:**
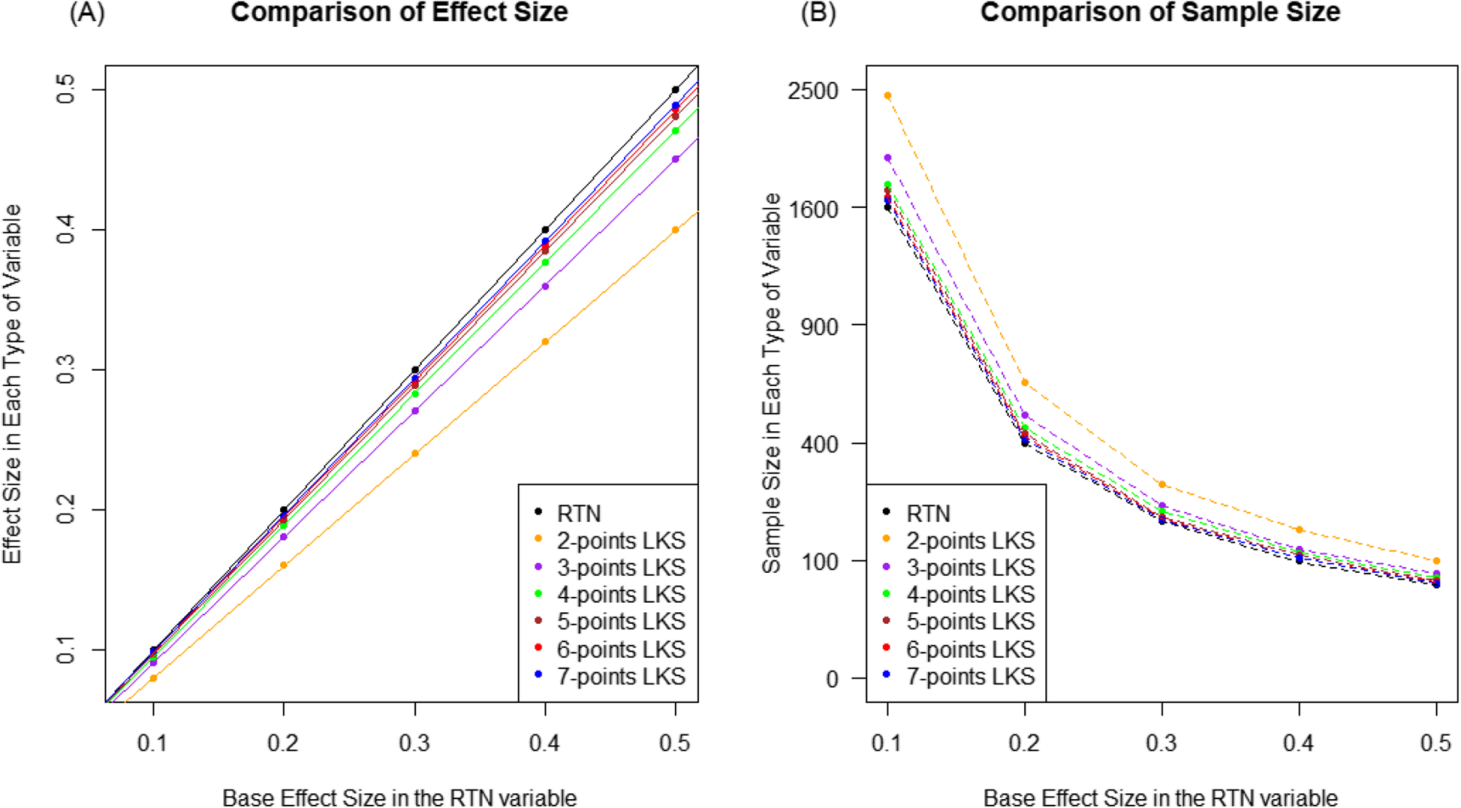
Effect size and required sample size according to the types of RTN and LKS variables. **A** Estimated effect sizes compared to that of the RTN variable. **B** Estimated required sample sizes to have 80% power for each effect size. Y-axis was drawn by square root transformed scale.

Based on the above results, we generalized the effects of the two factors using more systematically extended simulation settings. Specifically, we set five non-zero effect sizes for the RTN variable and then converted the RTN variable into LKS variables, while estimating their effect sizes. As shown in Fig. 2(A), the estimated effect sizes in each type of LKS variable were on a single line, indicating that the relative ratios of effect sizes between the LKS and RTN variables were consistent, regardless of the effect size in the RTN variable. Additionally, the relative ratios increased as the number of points in the LKS variable increased. Specifically, the ratios of the effect sizes between the two-point LKS and RTN variables were approximately 0.8 across all effect size settings, but they were over 95% for the LKS variable with five or more points. In summary, information loss of the LKS variable compared to the RTN variable depends only on the types of the LKS variable, and the five or more points LKS variables may provide statistical power comparable to that of the RTN variable. Subsequently, we focused on the sample sizes required to achieve a statistical power of 80% and compared them for the LKS and RTN variables. As shown in Fig. 2(B), when the effect sizes between the two groups was 0.1 in the RTN variable, 1,600 samples per group were required to achieve the statistical power, while 2,455 samples per group were required for the two-point LKS variable, but the five or more points LKS variable required additional sample sizes of less than 10% compared to those of the RTN variables. Moreover, as the effect sizes increased linearly, the required sample size decreased in the squared order [29]. This relationship was found for all types of variables, indicating that the ratios of the required sample sizes between the types of responses are consistent. As shown in Table 1 and Fig. 1, the results of the LKS variable were closer to those of the RTN variable when the points of the LKS variable increased.

Afterward, we additionally examined the characteristics of the merged LKS variables and compared them with those of the merged RTN variables. Based on the settings explained in the method section, we calculated the statistical power and the required sample size for the LKS variable to match the statistical power of the RTN variable, and summarized the results in Table 2.

**Table 2:** Power analysis of two sample t-test with the multiple correlated RTN and LKS variables. Proportions (%) of significantly identified sets and their standard errors were provided. Required sample sizes were also presented in the parenthesis.

Number of merged variables	Type of variable	Pairwise correlations between responses				
		0	0.25	0.5	0.75	1
2	RTN	50.51$\pm$0.50	42.44$\pm$0.49	36.24$\pm$0.48	31.71$\pm$0.47	29.19$\pm$0.45
	2-pt LKS	35.62$\pm$0.48 (152)	31.19$\pm$0.46 (140)	27.74$\pm$0.45 (136)	24.83$\pm$0.43 (132)	21.81$\pm$0.41 (148)
	3-pt LKS	42.74$\pm$0.49 (123)	36.73$\pm$0.48 (120)	32.20$\pm$0.47 (114)	28.53$\pm$0.45 (113)	24.58$\pm$0.43 (127)
	4-pt LKS	45.96$\pm$0.50 (111)	38.99$\pm$0.49 (111)	34.16$\pm$0.47 (109)	30.21$\pm$0.46 (106)	26.46$\pm$0.44 (113)
	5-pt LKS	47.93$\pm$0.50 (108)	40.39$\pm$0.49 (107)	34.66$\pm$0.48 (106)	30.24$\pm$0.46 (105)	27.33$\pm$0.45 (110)
	6-pt LKS	48.42$\pm$0.50 (106)	40.88$\pm$0.49 (106)	34.99$\pm$0.48 (105)	31.12$\pm$0.46 (102)	27.78$\pm$0.45 (107)
	7-pt LKS	49.32$\pm$0.50 (105)	41.39$\pm$0.49 (105)	35.45$\pm$0.48 (104)	31.18$\pm$0.46 (102)	28.10$\pm$0.45 (105)
3	RTN	67.75$\pm$0.47	50.66$\pm$0.50	40.47$\pm$0.49	33.90$\pm$0.47	29.19$\pm$0.45
	2-pt LKS	49.17$\pm$0.50 (154)	39.13$\pm$0.49 (137)	32.42$\pm$0.47 (131)	26.79$\pm$0.44 (130)	21.81$\pm$0.41 (148)
	3-pt LKS	58.41$\pm$0.49 (123)	45.38$\pm$0.50 (115)	36.35$\pm$0.48 (111)	30.90$\pm$0.46 (111)	24.58$\pm$0.43 (127)
	4-pt LKS	62.23$\pm$0.48 (113)	47.45$\pm$0.50 (107)	38.49$\pm$0.49 (106)	32.35$\pm$0.47 (106)	26.46$\pm$0.44 (113)
	5-pt LKS	64.66$\pm$0.48 (108)	48.94$\pm$0.50 (105)	39.25$\pm$0.49 (105)	32.98$\pm$0.47 (105)	27.33$\pm$0.45 (110)
	6-pt LKS	65.07$\pm$0.48 (106)	49.48$\pm$0.50 (104)	39.40$\pm$0.49 (104)	32.85$\pm$0.47 (103)	27.78$\pm$0.45 (107)
	7-pt LKS	65.69$\pm$0.47 (104)	49.73$\pm$0.50 (101)	40.04$\pm$0.49 (101)	33.31$\pm$0.47 (102)	28.10$\pm$0.45 (105)
5	RTN	87.25$\pm$0.33	58.92$\pm$0.49	44.11$\pm$0.50	34.58$\pm$0.48	29.19$\pm$0.45
	2-pt LKS	70.74$\pm$0.45 (153)	49.27$\pm$0.50 (125)	37.22$\pm$0.48 (122)	29.28$\pm$0.46 (122)	21.81$\pm$0.41 (148)
	3-pt LKS	80.21$\pm$0.40 (121)	55.10$\pm$0.50 (108)	41.32$\pm$0.49 (108)	32.63$\pm$0.47 (107)	24.58$\pm$0.43 (127)
	4-pt LKS	83.16$\pm$0.37 (112)	57.13$\pm$0.49 (104)	42.09$\pm$0.49 (105)	33.47$\pm$0.47 (104)	26.46$\pm$0.44 (113)
	5-pt LKS	84.90$\pm$0.36 (107)	57.81$\pm$0.49 (103)	42.76$\pm$0.49 (104)	33.90$\pm$0.47 (103)	27.33$\pm$0.45 (110)
	6-pt LKS	85.84$\pm$0.35 (105)	58.26$\pm$0.49 (102)	42.98$\pm$0.50 (103)	34.08$\pm$0.47 (103)	27.78$\pm$0.45 (107)
	7-pt LKS	85.96$\pm$0.35 (103)	58.38$\pm$0.49 (101)	43.43$\pm$0.50 (101)	34.54$\pm$0.48 (101)	28.10$\pm$0.45 (105)
10	RTN	99.34$\pm$0.08	69.31$\pm$0.46	47.85$\pm$0.50	36.16$\pm$0.48	29.19$\pm$0.45
	2-pt LKS	94.33$\pm$0.23 (153)	61.88$\pm$0.49 (118)	42.78$\pm$0.49 (115)	31.70$\pm$0.47 (120)	21.81$\pm$0.41 (148)
	3-pt LKS	97.80$\pm$0.15 (121)	66.34$\pm$0.47 (107)	45.91$\pm$0.50 (107)	35.04$\pm$0.48 (108)	24.58$\pm$0.43 (127)
	4-pt LKS	98.68$\pm$0.11 (113)	67.37$\pm$0.47 (104)	46.67$\pm$0.50 (103)	35.44$\pm$0.48 (103)	26.46$\pm$0.44 (113)
	5-pt LKS	98.96$\pm$0.10 (108)	68.13$\pm$0.47 (103)	47.68$\pm$0.50 (103)	35.54$\pm$0.48 (103)	27.33$\pm$0.45 (110)
	6-pt LKS	99.03$\pm$0.10 (106)	68.50$\pm$0.46 (102)	47.38$\pm$0.50 (102)	35.82$\pm$0.48 (103)	27.78$\pm$0.45 (107)
	7-pt LKS	99.07$\pm$0.10 (104)	68.53$\pm$0.46 (102)	47.79$\pm$0.50 (103)	35.83$\pm$0.48 (103)	28.10$\pm$0.45 (105)

We assumed that effect size between the two groups was 0.2 and sample size per group was 100.

*pt* point

As shown in Table 2, the following trends were observed: First, when the number of merged variables was fixed, the pairwise correlation was inversely associated with the statistical power. Specifically, regardless of the variable type, the statistical power was highest at R = 0 and lowest at R = 1. These results were derived from the positive pairwise correlation, which increased the standard error of the mean difference, thereby decreasing the test statistic and increasing the *p*-value. Second, when the correlation between the variables was fixed, the statistical power increased as the number of merged variables increased, except when R = 1. This was because the number of pairwise correlations between the variables increased faster than the number of variances of the variables. For example, when there were two variables, both the number of pairwise correlations and the number of variances were two; however, these values were 90 and 10, respectively, when there were 10 variables. Therefore, the relative influence of the pairwise correlation on the standard error of the mean difference increases as the number of merged variables increases. Third, across all settings, the LKS variable with five or more points required an additional sample size of less than 10% compared to the sample size used in the RTN variables. This means that these types of variables had slightly lower but comparable efficiencies to obtain the same statistical power. Moreover, when the type of LKS variable was fixed, the required sample size was the largest at R = 0 or 1 and relatively small in the other correlation settings. This might be due to a decrease in the pairwise correlation during the conversion of the RTN variable into the LKS variable, which decreased the standard error and relatively improved statistical power compared to the results with R = 0 or 1.

Next, we additionally examined the extent to which the pairwise correlation was preserved during the transformation from the RTN to LKS variables. Specifically, we assessed preservation using the goodness-of-fit measures employed in the above two analysis methods [23]. Among the measures, we selected the GFI and SRMR [28], and Table 3 presents these two values for each LKS variable type. To align with the results in Table 2, we used the same settings for pairwise correlation (except R = 1) and number of variables.

**Table 3:** Goodness of fit measures (GFI/SRMR) between correlation matrices from the RTN and LKS variables. Their means and standard errors were provided.

Number of merged variables	Type of variable	Pairwise correlations between responses			
		0	0.25	0.5	0.75
2	2-pt LKS	0.4090$\pm$0.0042 /0.0268$\pm$0.0002	0.8057$\pm$0.0020 /0.0521$\pm$0.0003	0.8373$\pm$0.0009 /0.0939$\pm$0.0003	0.8070$\pm$0.0007 /0.1190$\pm$0.0003
	3-pt LKS	0.5134$\pm$0.0042 /0.0190$\pm$0.0001	0.9264$\pm$0.0010 /0.0288$\pm$0.0002	0.9454$\pm$0.0004 /0.0494$\pm$0.0002	0.9123$\pm$0.0003 /0.0697$\pm$0.0002
	4-pt LKS	0.5969$\pm$0.0041 /0.0144$\pm$0.0001	0.9638$\pm$0.0006 /0.0192$\pm$0.0001	0.9768$\pm$0.0002 /0.0301$\pm$0.0002	0.9616$\pm$0.0002 /0.0415$\pm$0.0001
	5-pt LKS	0.6543$\pm$0.0040 /0.0115$\pm$0.0001	0.9793$\pm$0.0004 /0.0139$\pm$0.0001	0.9883$\pm$0.0001 /0.0203$\pm$0.0001	0.9808$\pm$0.0001 /0.0274$\pm$0.0001
	6-pt LKS	0.6947$\pm$0.0038 /0.0098$\pm$0.0001	0.9859$\pm$0.0003 /0.0112$\pm$0.0001	0.9933$\pm$0.0001 /0.0149$\pm$0.0001	0.9892$\pm$0.0001 /0.0196$\pm$0.0001
	7-pt LKS	0.7333$\pm$0.0037 /0.0083$\pm$0.0001	0.9900$\pm$0.0003 /0.0093$\pm$0.0001	0.9956$\pm$0.0001 /0.0117$\pm$0.0001	0.9935$\pm$0.0001 /0.0146$\pm$0.0001
3	2-pt LKS	0.3622$\pm$0.0035 /0.0378$\pm$0.0002	0.8259$\pm$0.0011 /0.0694$\pm$0.0002	0.8556$\pm$0.0005 /0.1184$\pm$0.0002	0.8267$\pm$0.0004 /0.1475$\pm$0.0003
	3-pt LKS	0.5421$\pm$0.0035 /0.0270$\pm$0.0001	0.9367$\pm$0.0005 /0.0395$\pm$0.0002	0.9501$\pm$0.0002 /0.0639$\pm$0.0002	0.9203$\pm$0.0002 /0.0867$\pm$0.0001
	4-pt LKS	0.6770$\pm$0.0031 /0.0204$\pm$0.0001	0.9689$\pm$0.0003 /0.0264$\pm$0.0001	0.9782$\pm$0.0001 /0.0396$\pm$0.0001	0.9646$\pm$0.0001 /0.0520$\pm$0.0001
	5-pt LKS	0.7612$\pm$0.0027 /0.0163$\pm$0.0001	0.9821$\pm$0.0002 /0.0195$\pm$0.0001	0.9887$\pm$0.0001 /0.0272$\pm$0.0001	0.9818$\pm$0.0001 /0.0349$\pm$0.0001
	6-pt LKS	0.8200$\pm$0.0024 /0.0136$\pm$0.0001	0.9882$\pm$0.0001 /0.0156$\pm$0.0001	0.9932$\pm$0.0000 /0.0202$\pm$0.0001	0.9897$\pm$0.0000 /0.0250$\pm$0.0001
	7-pt LKS	0.8565$\pm$0.0021 /0.0117$\pm$0.0000	0.9917$\pm$0.0001 /0.0129$\pm$0.0001	0.9956$\pm$0.0000 /0.0159$\pm$0.0001	0.9936$\pm$0.0000 /0.0190$\pm$0.0001
5	2-pt LKS	0.3176$\pm$0.0026 /0.0462$\pm$0.0001	0.8386$\pm$0.0006 /0.0825$\pm$0.0002	0.8734$\pm$0.0003 /0.1378$\pm$0.0002	0.8442$\pm$0.0003 /0.1713$\pm$0.0002
	3-pt LKS	0.5987$\pm$0.0024 /0.0327$\pm$0.0001	0.9371$\pm$0.0003 /0.0477$\pm$0.0001	0.9534$\pm$0.0001 /0.0751$\pm$0.0001	0.9266$\pm$0.0001 /0.1008$\pm$0.0001
	4-pt LKS	0.7592$\pm$0.0016 /0.0248$\pm$0.0001	0.9680$\pm$0.0001 /0.0320$\pm$0.0001	0.9785$\pm$0.0001 /0.0467$\pm$0.0001	0.9662$\pm$0.0001 /0.0607$\pm$0.0001
	5-pt LKS	0.8423$\pm$0.0011 /0.0200$\pm$0.0000	0.9806$\pm$0.0001 /0.0240$\pm$0.0001	0.9882$\pm$0.0000 /0.0324$\pm$0.0001	0.9823$\pm$0.0000 /0.0407$\pm$0.0001
	6-pt LKS	0.8894$\pm$0.0008 /0.0167$\pm$0.0000	0.9870$\pm$0.0001 /0.0190$\pm$0.0000	0.9926$\pm$0.0000 /0.0242$\pm$0.0001	0.9895$\pm$0.0000 /0.0295$\pm$0.0001
	7-pt LKS	0.9192$\pm$0.0006 /0.0143$\pm$0.0000	0.9907$\pm$0.0000 /0.0158$\pm$0.0000	0.9949$\pm$0.0000 /0.0192$\pm$0.0000	0.9933$\pm$0.0000 /0.0225$\pm$0.0000
10	2-pt LKS	0.2896$\pm$0.0017 /0.0523$\pm$0.0001	0.8344$\pm$0.0003 /0.0928$\pm$0.0001	0.8812$\pm$0.0002 /0.1536$\pm$0.0002	0.8562$\pm$0.0002 /0.1904$\pm$0.0002
	3-pt LKS	0.6375$\pm$0.0010 /0.0370$\pm$0.0000	0.9275$\pm$0.0002 /0.0537$\pm$0.0001	0.9506$\pm$0.0001 /0.0839$\pm$0.0001	0.9289$\pm$0.0001 /0.1119$\pm$0.0001
	4-pt LKS	0.7897$\pm$0.0006 /0.0281$\pm$0.0000	0.9601$\pm$0.0001 /0.0362$\pm$0.0000	0.9747$\pm$0.0000 /0.0523$\pm$0.0001	0.9651$\pm$0.0000 /0.0677$\pm$0.0001
	5-pt LKS	0.8639$\pm$0.0004 /0.0226$\pm$0.0000	0.9749$\pm$0.0001 /0.0269$\pm$0.0000	0.9849$\pm$0.0000 /0.0363$\pm$0.0001	0.9804$\pm$0.0000 /0.0455$\pm$0.0001
	6-pt LKS	0.9052$\pm$0.0003 /0.0189$\pm$0.0000	0.9828$\pm$0.0000 /0.0216$\pm$0.0000	0.9900$\pm$0.0000 /0.0272$\pm$0.0000	0.9877$\pm$0.0000 /0.0329$\pm$0.0000
	7-pt LKS	0.9305$\pm$0.0002 /0.0162$\pm$0.0000	0.9874$\pm$0.0000 /0.0179$\pm$0.0000	0.9929$\pm$0.0000 /0.0216$\pm$0.0000	0.9917$\pm$0.0000 /0.0251$\pm$0.0000

The sample size in each dataset was assumed to be 200.

*pt* point

In summary, the GFI value increased as the points of the LKS variable increased in all settings. However, in the R = 0 setting, the values did not exceed 0.95, which differed significantly from those in the other correlation settings. This is because the GFI calculated the relative improved similarity by comparing it with the identity correlation matrix and the RTN variables were also generated from the identity matrix. Consequently, the improved similarity was difficult to be high in this setting. However, in the other three correlation settings (R = 0.25, 0.5, and 0.75), all the GFI values significantly increased and exceeded 0.95, particularly in the LKS variable with four or more points. Subsequently, for SRMR, most values in the R = 0 setting were below 0.05. However, in the other three correlation settings, these values generally increased, staying below 0.05 only for the LKS variables with five or more points. Considering the results of these two measures, the LKS variables with five or more points exhibited desirable similarities to the RTN variables in terms of the correlation matrix during the transformation.

Finally, we examined the characteristics of the LKS variables when used as explanatory variables, as these variables may serve as predictors depending on their relationship with other variables in the study. After the analysis with the simulation settings explained in the method section was completed, we found several patterns in the results, as presented in Table 4. First, the statistical power increased as the number of explanatory variables increased in each combination of variable types. Second, the required sample size, to achieve the same statistical power as the results of the RTN explanatory variables, decreased as the number of explanatory variables increased. Third, as the number of points in the LKS variables increased, the required sample size decreased, reaching less than 220 for the five-point LKS variable. Combined with previous results, we concluded that the LKS variable with five or more points, not only as a response but also as an explanatory variable, may yield comparable results with those from the RTN variable.

**Table 4:** Power analysis of linear regression approach according to numbers of explanatory variables. Proportions (%) of significantly identified sets and their standard errors were provided. Required sample sizes were also presented in the parenthesis.

Type of Response	Type of Explanatory variable	Numbers of explanatory variables				
		1	2	13	5	10
RTN	RTN	80.22$\pm$0.40	92.11$\pm$0.27	95.62$\pm$0.20	97.84$\pm$0.15	98.48$\pm$0.12
	2-pt LKS	61.88$\pm$0.49 (308)	77.38$\pm$0.42 (298)	85.27$\pm$0.35 (290)	91.72$\pm$0.28 (274)	95.21$\pm$0.21 (244)
	3-pt LKS	72.08$\pm$0.45 (246)	87.34$\pm$0.33 (238)	92.29$\pm$0.27 (232)	95.78$\pm$0.20 (228)	97.27$\pm$0.16 (220)
	4-pt LKS	75.76$\pm$0.43 (222)	89.25$\pm$0.31 (220)	93.76$\pm$0.24 (218)	96.94$\pm$0.17 (214)	97.92$\pm$0.14 (210)
	5-pt LKS	76.98$\pm$0.42 (216)	90.07$\pm$0.30 (214)	94.37$\pm$0.23 (212)	96.98$\pm$0.17 (212)	98.10$\pm$0.14 (208)
2-pt LKS	RTN	61.45$\pm$0.49	75.70$\pm$0.43	81.28$\pm$0.39	85.83$\pm$0.35	86.88$\pm$0.34
	2-pt LKS	44.10$\pm$0.50 (314)	57.23$\pm$0.49 (298)	65.51$\pm$0.48 (282)	73.43$\pm$0.44 (264)	78.49$\pm$0.41 (240)
	3-pt LKS	52.71$\pm$0.50 (246)	67.70$\pm$0.47 (238)	74.60$\pm$0.44 (232)	80.53$\pm$0.40 (230)	83.47$\pm$0.37 (216)
	4-pt LKS	56.74$\pm$0.50 (226)	70.88$\pm$0.45 (222)	77.86$\pm$0.42 (218)	83.07$\pm$0.38 (214)	85.32$\pm$0.35 (208)
	5-pt LKS	58.55$\pm$0.50 (216)	72.70$\pm$0.45 (216)	78.47$\pm$0.41 (214)	84.16$\pm$0.37 (212)	85.97$\pm$0.35 (204)
3-pt LKS	RTN	71.69$\pm$0.45	85.32$\pm$0.35	90.41$\pm$0.29	94.13$\pm$0.24	94.74$\pm$0.22
	2-pt LKS	53.13$\pm$0.50 (310)	68.13$\pm$0.47 (298)	76.28$\pm$0.43 (286)	84.33$\pm$0.36 (270)	88.86$\pm$0.31 (240)
	3-pt LKS	63.12$\pm$0.48 (244)	78.92$\pm$0.41 (238)	84.96$\pm$0.36 (234)	90.29$\pm$0.30 (230)	92.62$\pm$0.26 (216)
	4-pt LKS	67.02$\pm$0.47 (224)	81.71$\pm$0.39 (222)	87.81$\pm$0.33 (220)	92.35$\pm$0.27 (220)	93.65$\pm$0.24 (208)
	5-pt LKS	68.22$\pm$0.47 (216)	82.53$\pm$0.38 (216)	88.33$\pm$0.32 (214)	92.86$\pm$0.26 (212)	94.17$\pm$0.23 (204)
4-pt LKS	RTN	75.21$\pm$0.43	88.89$\pm$0.31	93.12$\pm$0.25	95.96$\pm$0.20	96.57$\pm$0.18
	2-pt LKS	57.05$\pm$0.50 (310)	72.26$\pm$0.45 (298)	80.59$\pm$0.40 (288)	87.64$\pm$0.33 (268)	92.04$\pm$0.27 (240)
	3-pt LKS	66.93$\pm$0.47 (246)	82.61$\pm$0.38 (240)	88.33$\pm$0.32 (234)	93.18$\pm$0.25 (228)	95.04$\pm$0.22 (216)
	4-pt LKS	70.81$\pm$0.45 (220)	85.16$\pm$0.36 (220)	90.62$\pm$0.29 (220)	94.54$\pm$0.23 (214)	96.03$\pm$0.20 (208)
	5-pt LKS	72.35$\pm$0.45 (216)	86.19$\pm$0.35 (218)	91.45$\pm$0.28 (214)	94.97$\pm$0.22 (212)	96.13$\pm$0.19 (204)
5-pt LKS	RTN	77.28$\pm$0.42	89.94$\pm$0.30	93.87$\pm$0.24	96.66$\pm$0.18	97.34$\pm$0.16
	2-pt LKS	58.61$\pm$0.49 (308)	73.96$\pm$0.44 (298)	81.99$\pm$0.38 (288)	88.92$\pm$0.31 (268)	93.42$\pm$0.25 (240)
	3-pt LKS	69.14$\pm$0.46 (242)	84.59$\pm$0.36 (236)	90.21$\pm$0.30 (232)	94.19$\pm$0.23 (228)	95.89$\pm$0.20 (216)
	4-pt LKS	72.54$\pm$0.45 (222)	86.78$\pm$0.34 (222)	91.94$\pm$0.27 (220)	95.56$\pm$0.21 (214)	96.67$\pm$0.18 (208)
	5-pt LKS	74.01$\pm$0.44 (216)	87.74$\pm$0.33 (214)	92.58$\pm$0.26 (214)	95.76$\pm$0.20 (212)	96.69$\pm$0.18 (208)

The sample size in each dataset was assumed to be 200.

*pt* point

## Discussion

In this study, we investigated the statistical characteristics of the LKS variable when used as a continuous variable by comparing type 1 error, statistical power, and correlation similarity with those of the RTN variable in several specific situations as an example. From this assumption and the corresponding analysis results, we concluded that this approach was valid, as claimed in previous research [16, 17]. Additionally, using the LKS variable with five or more points as a continuous variable in the factor analysis or structural equation modeling was found to be appropriate.

Specifically, we first found no substantial violation in the type 1 error rate, regardless of the variable type. Therefore, significant results obtained from the continuous approach with the LKS variable are reliable. Second, we considered the statistical power given the single response between the two groups. As mentioned in the existing research [17], we confirmed that the statistical power of the LKS variable became closer to that of the RTN variable as the points of the LKS variable increased. In addition to the existing results, we found that the decreased rate of effect size compared with that of the RTN variable primarily depended on the type of LKS variable. At the five or more points LKS variable, the decreased rates of effect size remained below 5%, and the additionally required sample sizes were less than 10% compared to those of the RTN variables. Although these types of LKS variables have already been widely used [18], we encourage their use based on their statistical properties with minimal concern. Even in the lower-point types of the LKS variable, this does not result in substantial problems, except for low statistical power.

Third, merging multiple variables into a variable of the total score has been widely used, particularly in clinical studies with tools measuring mental health, such as depression [30], cognitive function [31], and addiction [32]. We found that the merging approach improved the statistical power; however, an inverse relationship was observed between the pairwise correlation of responses and the amount of improved statistical power. This indicates that moderate reliability among the responses may improve the statistical power more than high reliability. Therefore, we can infer the improvement of statistical power by calculating pairwise correlations or Cronbach’s $\alpha$ [33] before the analysis. Additionally, concerning factor analysis or structural equation modeling that uses correlated multiple responses without merging, we examined the correlation change during the transformation from the RTN variable to the LKS variables. In summary, the LKS variable with five or more points had a reasonably similar correlation matrix compared to that of the RTN variable. This indicates that these types of LKS variables may provide comparable results in these two methods to those of their continuous latent variables. Consequently, when using statistical analysis programs, we suggest treating LKS variables with four or less points as ordinal variables based on the probit assumption [34] and LKS variables with five or more points as continuous variables.

Fourth, we considered a situation in which the LKS variables were used as explanatory variables. In this case, the required sample size was consistent for each type of explanatory variable, regardless of the type of response. Therefore, combined with the previous simulation results, we concluded that the use of the five or more points LKS variable as a continuous variable did not result in a significant loss of statistical power in either response or explanatory variables. In addition, as shown in Table 4, a larger number of associated LKS variables may achieve statistical power closer to that of the RTN variables. This suggests that the LKS variables can be used to construct prediction or classification models that contain numerous explanatory variables.

Finally, as a limitation, we mention that generalization of our analysis results with the assumption of the RTN distribution needs to be careful. Although the assumption has reasonable statistical properties not only that the median of this distribution has higher density than its periphery, but also that floor and ceiling effects can be naturally taken into account, and consequently skewness may also be considered as the distribution shifts. However, real data may have more diverse forms of distributions in fact. Therefore, detailed results may change for different continuous latent distributions, even if the main conclusion remain similar. Future research that relaxes the assumption with nonparametric concepts may be required.

## Conclusions

Investigating the LKS variables as continuous variables using parametric approaches confirmed that this type of variable generally maintained a type 1 error rate. In addition, LKS variables with five or more points exhibited statistical power and correlation structures comparable to those of the assumed RTN variables. The simulation also included settings where multiple LKS variables were merged as a response, incorporated based on their correlation structures, or employed as explanatory variables. From the simulation analysis, we concluded that the LKS variable can be applied using the same parametric approaches that were developed for continuous variables, such as t-test, linear regression, factor analysis, structural equation modeling, and prediction model construction approaches. This finding supports the reliability of parametric statistical methods when treating LKS variables as continuous.

## Data Availability

No datasets were generated or analysed during the current study.

## Abbreviations

LKS: Likert scale
RTN: Rectified normal distribution
GFI: Goodness of fit index
SRMR: Standardized root mean squared residual
